# Recombinant viral hemorrhagic septicemia virus with rearranged genomes as vaccine vectors to protect against lethal betanodavirus infection

**DOI:** 10.3389/fimmu.2023.1138961

**Published:** 2023-03-14

**Authors:** Sandra Souto, Emilie Mérour, Alain Le Coupanec, Annie Lamoureux, Julie Bernard, Michel Brémont, Jean K. Millet, Stéphane Biacchesi

**Affiliations:** ^1^ Microbiology and Parasitology, Universidade de Santiago de Compostela, Santiago de Compostela, Spain; ^2^ Université Paris-Saclay, INRAE, UVSQ, Virologie et Immunologie Moléculaires, Jouy-en-Josas, France

**Keywords:** Novirhabdovirus, betanodavirus, VHSV, NNV, live attenuated vaccine, trout, sole

## Abstract

The outbreaks of viral hemorrhagic septicemia (VHS) and viral encephalopathy and retinopathy (VER) caused by the enveloped novirhabdovirus VHSV, and the non-enveloped betanodavirus nervous necrosis virus (NNV), respectively, represent two of the main viral infectious threats for aquaculture worldwide. Non-segmented negative-strand RNA viruses such as VHSV are subject to a transcription gradient dictated by the order of the genes in their genomes. With the goal of developing a bivalent vaccine against VHSV and NNV infection, the genome of VHSV has been engineered to modify the gene order and to introduce an expression cassette encoding the major protective antigen domain of NNV capsid protein. The NNV Linker-P specific domain was duplicated and fused to the signal peptide (SP) and the transmembrane domain (TM) derived from novirhabdovirus glycoprotein to obtain expression of antigen at the surface of infected cells and its incorporation into viral particles. By reverse genetics, eight recombinant VHSVs (rVHSV), termed NxGyCz according to the respective positions of the genes encoding the nucleoprotein (N) and glycoprotein (G) as well as the expression cassette (C) along the genome, have been successfully recovered. All rVHSVs have been fully characterized *in vitro* for NNV epitope expression in fish cells and incorporation into VHSV virions. Safety, immunogenicity and protective efficacy of rVHSVs has been tested *in vivo* in trout (*Oncorhynchus mykiss)* and sole (*Solea senegalensis*). Following bath immersion administration of the various rVHSVs to juvenile trout, some of the rVHSVs were attenuated and protective against a lethal VHSV challenge. Results indicate that rVHSV N2G1C4 is safe and protective against VHSV challenge in trout. In parallel, juvenile sole were injected with rVHSVs and challenged with NNV. The rVHSV N2G1C4 is also safe, immunogenic and efficiently protects sole against a lethal NNV challenge, thus presenting a promising starting point for the development of a bivalent live attenuated vaccine candidate for the protection of these two commercially valuable fish species against two major diseases in aquaculture.

## Introduction

Nervous necrosis virus (NNV) is the causative agent of viral nervous necrosis (VNN), a devastating neurological disease, also known as viral encephalopathy and retinopathy (VER). The most common clinical signs of NNV infection are abnormal swimming behavior, loss of appetite and changes in fish coloration. Lesions observed in NNV-infected fish clearly demonstrate its marked neurotropism, the virus preferentially infects nerve cells, especially those of the central nervous system and retina (1). NNV has been isolated from a wide range of both marine and freshwater fish species and is responsible for significant losses in aquaculture industry as mortality rates of up to 100% are observed in larvae and juveniles infected by the virus (2). This small (~30 nm diameter), spherical, non-enveloped virus belongs to the *Betanodavirus* genus in the *Nodaviridae* family (3). Its genome contains two single-stranded positive-sense RNA molecules of approximately 3.1 kb (RNA1) and 1.4 kb (RNA2). An additional nonencapsidated subgenomic RNA coterminal with the 3’ end of RNA1 (RNA3, ~0.4 kb) is transcribed during virus replication. The three RNAs are capped at their 5’ ends but lack poly(A) tails at their 3’ ends (4). RNA1 encodes the viral RNA-dependent RNA polymerase (RdRp, 110 kDa), whereas RNA2 encodes the capsid protein (40-42 kDa) and RNA3 encodes two small non-structural proteins B1 and B2, which have antagonistic effects on cell survival (5).

The NNV isolates have been classified into four genotypes: striped jack nervous necrosis virus (SJNNV), tiger puffer nervous necrosis virus (TPNNV), redspotted grouper nervous necrosis virus (RGNNV) and barfin flounder nervous necrosis virus (BFNNV), based on a small highly variable sequence of RNA2, the so-called T4 region (6). Furthermore, these four genotypes group into three distinct serotypes A, B, and C with RGNNV belonging to serotype C (7). RGNNV has been the predominant genotype in Europe, although the emergence of reassortant strains, between the RGNNV and SJNNV genotypes, has undergone a dramatic expansion in the past few decades in the South of Europe (8–10) posing a significant risk to the cultivation of species of great economic importance such as gilthead sea bream (*Sparus aurata*), turbot (*Scophthalmus maximus*), European sea bass (*Dicentrarchus labrax*) and Senegalese sole (*Solea senegalensis*) (11–13). Vaccination is considered crucial for VER prevention and control since no effective treatments are available for this disease in aquaculture. A significant number of new experimental vaccines for this virus have been described in recent years, yet only two are commercialized in Europe, based on an inactivated RGNNV strain delivered by injection. Thus, both vaccines are restricted to the RGNNV genotype and one fish species (European sea bass). Therefore, the search for a more effective immunization system that covers a wider number of species and genotypes is still pending.

Vaccines based on inactivated viruses are usually very safe, but immune responses elicited are generally different from that produced by the live pathogen. In contrast, the use of attenuated live viruses has led to better responses but has raised safety concerns about the possibility of reversion to a virulent phenotype. Viruses from different families have been genetically engineered to develop vector-based vaccines aimed at protecting against viral diseases. The viral vectors frequently used include vaccinia virus (*Poxviridae*), Venezuelan equine encephalitis virus (*Togaviridae*), human adenovirus (*Adenoviridae*), Sendai virus (*Paramyxoviridae*), and vesicular stomatitis virus (*Rhabdoviridae*). Rhabdoviruses (14) represent promising platforms for developing novel vaccines (15–19) because they have been shown to be an effective means for heterologous antigen expression *in vivo* due to their high carrying capacity and genomic stability (20). A member of this family, the viral hemorrhagic septicemia virus (VHSV), is the causative agent of a very contagious and acute systemic disease leading to high mortality in a large panel of wild and commercial fish species worldwide (21). VHSV is listed as notifiable by the World Organization for Animal Health (WOAH/OIE). VHSV is considered as a serious economic and social threat for fish farms with significant environmental impact on natural resources. VHSV has been isolated from more than 82 different freshwater and marine species throughout the Northern Hemisphere, including North America, Asia, and Europe, including rainbow trout (*Oncorhynchus mykiss*), turbot, sea bass and sole (22). This virus is enveloped and its genome consists of a non-segmented negative-sense single-stranded RNA molecule of about 11 kilobases which encode six proteins in the order 3’-N-P-M-G-NV-L-5’ (23, 24). The viral RNA encodes five structural proteins. A nucleoprotein (N) which tightly encapsidates viral genome and antigenome RNAs together with a polymerase-associated phosphoprotein (P) and the large RNA-dependent RNA polymerase (L), hence forming a helical ribonucleoprotein complex (RNP). The matrix protein (M) interacts with the RNP and the viral envelope where the unique viral surface glycoprotein (G) is inserted by its transmembrane (TM) domain. In contrast to other rhabdoviruses, the VHSV genome possesses an additional gene, located between the G and L genes, that encodes a small non-structural NV protein essential for host innate immunity evasion (25–28). Due to the presence of the NV gene, VHSV is classified together with infectious hematopoietic necrosis virus (IHNV) in the genus *Novirhabdovirus*.

For Rhabdoviruses, the gene order is crucial for virus replication due to a decreasing gradient of transcription from the 3’ to the 5’ end. The viral polymerase binds to the 3’ end of the genome and starts transcription in a sequential gene-start-gene-end mechanism resulting in one mRNA species for each viral gene (29–32). Between each gene, the polymerase can dissociate from the genome, resulting in a gradient of expression in which the 3’ proximal genes are more transcribed than those located at the 5’ end. The modification of the gene order has an important impact on virus replication and pathogenicity as demonstrated by Wertz and colleagues on vesicular stomatitis virus (VSV) (33, 34) and our group on IHNV (19). In both cases, the N gene position seems to be one of the most critical factors for viral pathogenicity. Indeed, decreasing the amount of N protein by moving N gene downstream along the genome delayed the kinetics of replication and increased interferon expression leading to an attenuated phenotype (19, 35). These recombinant viruses were less pathogenic but maintained their immunogenicity *in vivo* due in part to the concomitant upstream displacement of the G gene within the genome allowing for increased expression of the G protein, the main target for neutralizing antibodies (19, 33–37). These data demonstrate that moving the N and G genes along the rhabdovirus genome is a promising approach for vaccine development in fish and mammals.

In this study, we develop a strategy to produce a live-attenuated vaccine against both VHS and VER by engineering the genome of VHSV to modify the gene order and to introduce an expression cassette encoding the major protective antigenic domain of NNV capsid protein allowing its incorporation into VHSV virions. Eight recombinant VHSVs (rVHSV), termed NxGyCz according to the respective positions of the genes encoding the nucleoprotein (N) and glycoprotein (G) as well as the expression cassette (C) along the genome, have been produced and tested for their safety, immunogenicity and protective efficacy in two fish species.

## Results

### Characterization of recombinant VHSVs expressing NNV epitopes

**Figure 1:**
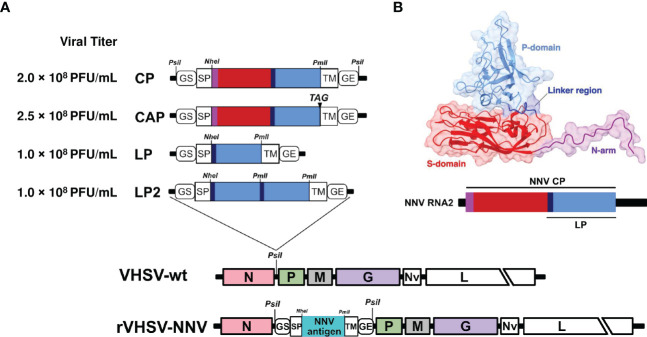
Construction of recombinant VHSV vectors expressing NNV capsid or capsid domains. **(A)** Schematic representation of NNV epitopes inserted in VHSV expression cassette driving antigen expression at the cell surface and incorporation in recombinant virus particles. The cassette is located between N and P genes in the VHSV genome. GS; gene start, GE; gene end, SP; signal peptide and TM; transmembrane domain (both SP and TM are derived from VHSV G). Full-length NNV capsid (CP or CAP including the stop codon TAG) or domains of CP (LP and LP2; P-domain plus linker region in single copy or in duplicate, respectively) were cloned in VHSV expression cassette. Unique enzyme restriction sites used in those constructs are indicated. **(B)** Structure of nervous necrosis virus (NNV) capsid protein. Ribbon diagram with surfaces displayed of a capsid subunit based on the PDB structure 4WIZ. Molecular visualizations were performed using Chimera X. Each capsid subunit is composed of three main domains: the N-terminal arm (N-arm), shown in purple (residues at the N-terminal extremity of the N-arm are not shown since they were found to be disordered), the shell domain (S-domain), shown in red, and the protrusion domain (P-domain), shown in light blue. The linker region, shown in blue, connects the S-domain with the P-domain. Below is a representation of the NNV genomic RNA2 with the regions encoding the different domains of the capsid.

Recombinant rVHSVs expressing NNV capsid or capsid domains were generated as described previously (18, 38), using the expression cassette inserted in the non-coding region between N and P genes. The encoding nucleotide sequences of the full-length NNV capsid or derived domains, which are known to be highly immunogenic (7) were cloned in this expression cassette in fusion with the signal peptide (SP) sequence derived from the IHNV glycoprotein G gene, and the transmembrane sequence (TM) from VHSV G gene. The expression cassettes were flanked with the gene start and gene end signals of VHSV in order to be recognized by the viral polymerase and to direct the efficient expression of heterologous genes (**Figure 1A**). Four recombinant viruses were produced expressing a membrane-targeted capsid protein (CP), a secreted form of the capsid protein (CAP), the linker region and the protruding domain (LP), which contained the major protective epitopes against NNV (39, 40) or a duplication of this LP domain (LP2) (**Figures 1A, B**). All recombinant viruses were readily recovered using the established reverse genetics system for VHSV (27). Recombinant viruses were amplified through 2 passages in fish EPC cells. Titers reached 2 × 10^8^ PFU/mL for rVHSV-CP, 2.5 × 10^8^ PFU/mL for rVHSV-CAP and 1 × 10^8^ PFU/mL for both rVHSV-LP and rVHSV-LP2 (**Figure 1A**).

**Figure 2:**
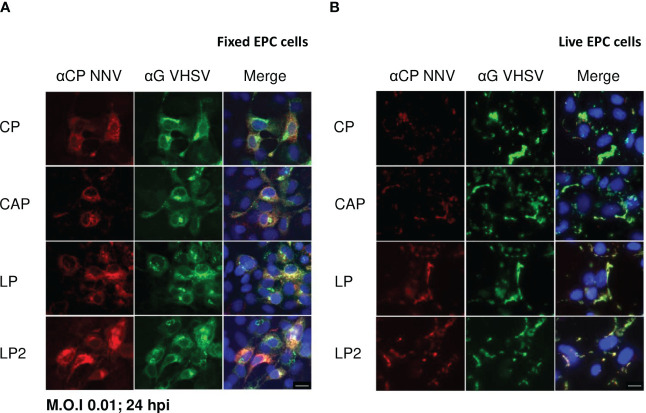
Expression of NNV antigens in rVHSVs infected cells. The expression of NNV antigens was assessed by indirect immunofluorescence assays on EPC cells. The cells were infected with rVHSV-CP, rVHSV-CAP, rVHSV-LP or rVHSV-LP2 at an MOI of 0.01 and incubated at 14 ˚C. **(A)** At 24 h post-infection, cells were fixed and permeabilized with alcohol/acetone and NNV and VHSV G expression were detected using a pAb against NNV (red) and a mAb against VHSV G (green), respectively. **(B)** At 24 h post-infection, membrane expression of NNV antigens was visualized on live cells in PBS using the same antibodies. Nuclei were stained with Hoechst (blue). Bars, 10 µm.

To assess the expression of NNV antigens by rVHSVs, EPC cells were infected with each recombinant virus at a multiplicity of infection (MOI) of 0.01. At 24 h post-infection, the expression of the NNV capsid protein or capsid domains was evaluated by indirect immunofluorescence on fixed or live infected cells (**Figure 2**). All recombinant viruses enabled the expression of the capsid protein or capsid domains in the cytoplasm of infected EPC cells, as shown by the co-labelling with anti-VHSV G mAb and anti-NNV pAb (**Figure 2A**). Next, we analyzed the expression of NNV capsid in live infected EPC cells to ensure the correct routing of the antigen along the secretory pathway towards the plasma membrane (**Figure 2B**). All recombinant viruses expressed and correctly addressed NNV capsid or capsid domains at the surface of infected EPC cells, a pattern of expression similar to that observed for VHSV G. The secreted form of the NNV capsid protein was also detected at the surface of the infected cells probably due to its propensity to interact with lipid bilayers (41).

**Figure 3:**
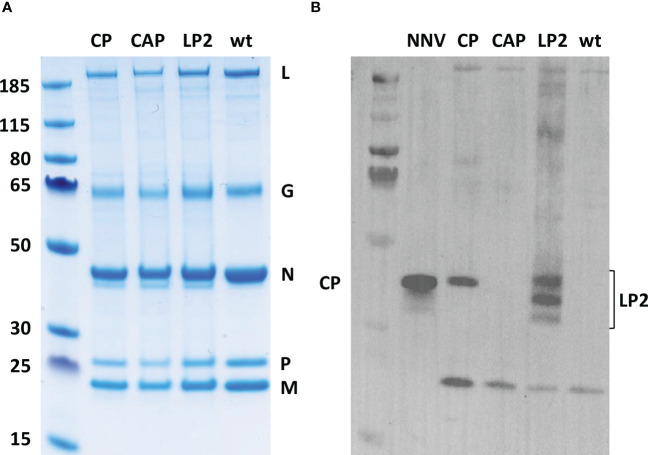
Analysis of NNV antigens incorporation in recombinant virus particles. **(A)** Six micrograms of sucrose-purified viral proteins were separated on a 4-12% polyacrylamide gel and stained by Coomassie blue. **(B)** Two micrograms of sucrose-purified viral proteins were denatured, loaded and migrated on an SDS page gel. The gel was electrotransferred onto a PVDF membrane and NNV antigens were detected with a rabbit pAb directed against NNV.

The incorporation efficiency of heterologous antigens at the surface of recombinant Novirhabdoviruses with an expression cassette inserted between N and P genes has been previously demonstrated in the laboratory (18, 38). The NNV antigen could not be clearly visualized on SDS-PAGE after Coomassie blue staining as it co-migrated with the N of VHSV (**Figure 3A**). We therefore validated the expression of the NNV antigen at the surface of the VHSV platforms by Western-blot assay on sucrose-purified viruses. **Figure 3B** shows that rVHSV-CP and rVHSV-LP2 both express the NNV antigen at the expected size (43 kDa and 35 kDa, respectively). Other forms of LP2 antigens (around 40 and 30 kDa) were also detected and are likely due to as yet uncharacterized post-translational modifications of this domain. In contrast, no NNV antigen was detected in rVHSV-CAP virions which express a secreted form of NNV capsid without VHSV G-derived TM domain, thus demonstrating the specificity of the NNV antigen incorporation into rVHSV particles.

### Recovery of rVHSVs with rearranged gene order

**Figure 4:**
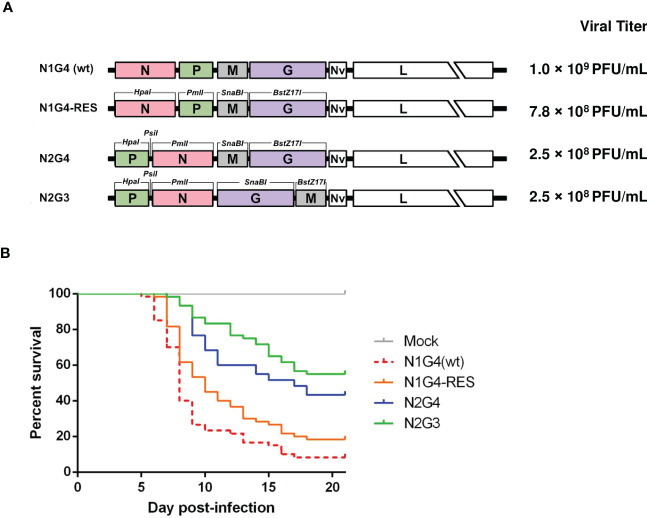
VHSV genome rearrangement. **(A)** Schematic representation of the engineered rVHSV genomes with rearranged gene order. Restriction enzyme sites inserted by site-directed mutagenesis at the beginning and the end of the N, P, M, G and NV ORF are indicated on the N1G4-RES genome. **(B)** INRA synthetic strain virus-free juvenile rainbow trout (n = 50 per group, mean weight 1.8 g) were infected by bath immersion with rVHSVs as indicated (final titer, 5 × 10^4^ PFU/mL) for 2 h at 10°C. Mortality rates were recorded daily and is presented as percent of survival.

Unique restriction enzyme sites were introduced by site-directed mutagenesis immediately upstream and downstream of the start and the stop codons of each ORF in the VHSV genome. Restriction enzyme sites were *HpaI* for N gene, *PmlI* for P gene, *SnaBI* for M gene, *BstZ17I* for G gene and *PmeI* for the NV gene, respectively (**Figure 4A**). The recombinant viruses were readily recovered as previously described (19, 27). Recombinant viruses with rearranged gene order were named according to their respective N and G gene position: N1G4 (3’-N-P-M-G-NV-L-5’) which corresponds to the recombinant virus that contains additional restriction enzyme sites introduced to each ORF (designated RES) or the wild-type virus (wt), N2G3 (3’-P-N-G-M-NV-L-5’) and N2G4 (3’-P-N-M-G-NV-L-5’), (**Figure 4A**). Based on the data obtained for IHNV (19), two gene orders were directly tested for VHSV, N2G3 and N2G4, for which the balance between attenuation and immunogenicity was optimal for IHNV, in order to evaluate the effect of such approach for VHSV attenuation.

The pathogenicity of the rVHSV was assessed by infecting juvenile rainbow trout (mean weight of 1.8 g) with the selected viruses and mortality rates were recorded daily for up to 21 days. As shown in **Figure 4B**, a similar fish mortality rate was observed between N1G4(wt) and N1G4-RES. For both viruses, the mortality started at day 5-6 post infection and reached 82% to 92% of cumulative mortality, respectively, at day 21. These data indicated that the addition of 10 restriction enzyme sites in the VHSV genome has a limited effect on virus pathogenicity. In contrast, N2G4 and N2G3 were attenuated *in vivo* inducing only 57% and 45% of cumulative mortality, respectively, at day 21. This confirms that changing the gene order impacts pathogenicity.

### Characterization of rVHSVs expressing a duplication of NNV LP domain in a gene order attenuated backbone

**Figure 5:**
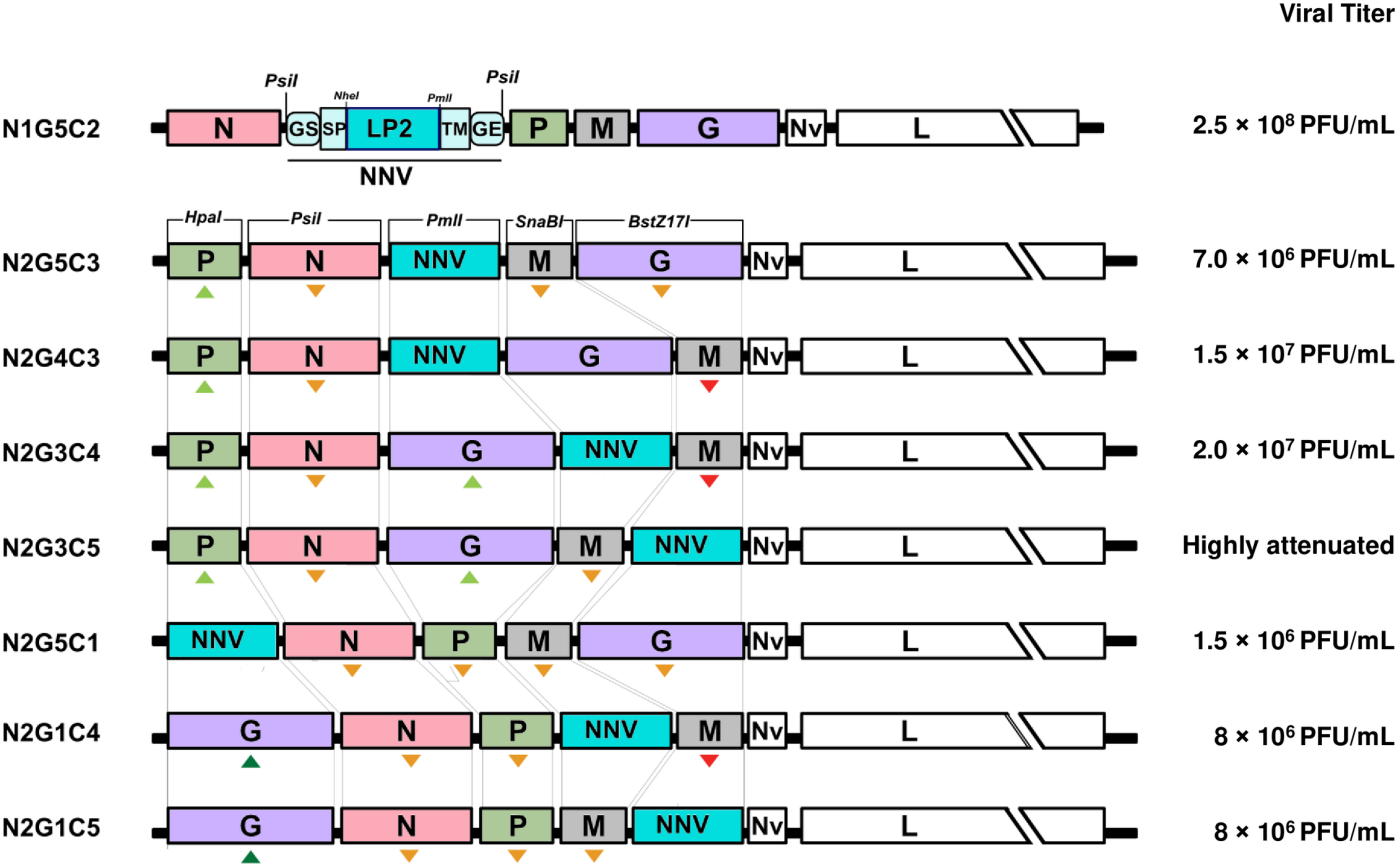
Recombinant VHSV expressing NNV epitopes with rearranged genome. Schematic representation of the engineered rVHSV genomes expressing NNV epitopes with rearranged gene order. Nine rVHSV, termed NxGyCz according to the respective positions of the genes encoding the nucleoprotein (N) and glycoprotein (G) as well as the expression cassette (C) along the genome. Viral titers (PFU/mL) obtained after two passages in EPC cells are indicated on the right.

Based on above results, the position of N and G genes along the VHSV genome has a great effect on VHSV virulence in trout. Therefore, N gene was kept at position 2 in order to maintain a basal level of attenuation. In parallel, the G gene and the NNV epitope expressing cassette were inserted at different positions along the VHSV genome to balance their levels of expression and thus their potential immunogenicity *in vivo*. Seven cDNA constructs were designed and termed NxGyCz according to the respective positions of N and G genes as well as the expression cassette C along the genome: N2G5C3, N2G4C3, N2G3C4, N2G3C5, N2G5C1, N2G1C4 and N2G1C5 (**Figure 5**). Based on three criteria: level of expression, level of incorporation in VHSV virion and high immunogenicity as duplication of NNV major epitope domain, the LP2 antigen was selected and inserted in these constructs. The recombinant viruses, rVHSV_GO_-NNV (with GO for modified gene order), were successfully recovered by reverse genetics and amplified in fish cells, except for N2G3C5 for which a total cytopathic effect (CPE) was never achieved. They reached titers ranging between 1.5 × 10^6^ PFU/mL to 2 × 10^7^ PFU/mL, but somewhat attenuated compared to the rVHSV N1G5C2 with the N gene in first position (2.5 × 10^8^ PFU/mL).

**Figure 6:**
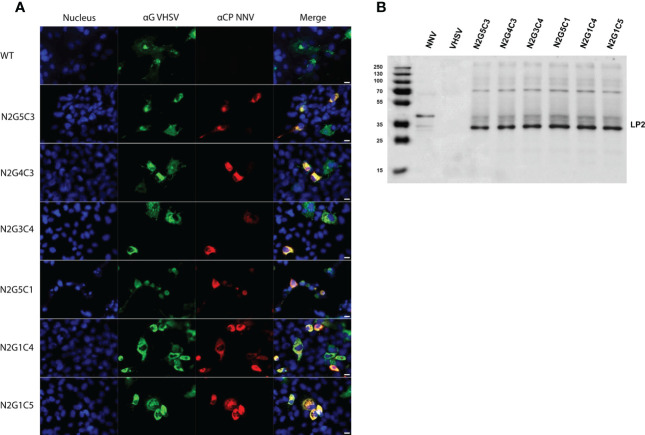
Characterization of rVHSV_GO_-NNV *in vitro* in fish cells and validation of NNV epitope incorporation in recombinant virus particles. **(A)** The expression of NNV antigens was assessed by indirect immunofluorescence assays on EPC cells. The cells were infected with rVHSV_GO_-NNV at an MOI of 0.1 and incubated at 14 ˚C. At 72 h post-infection, cells were fixed and permeabilized with alcohol/acetone and NNV and VHSV G expression were detected using a pAb against NNV (red) and a mAb against VHSV G (green), respectively. Nuclei were stained with Hoechst (blue). Bars, 10 µm. **(B)** Two micrograms of sucrose-purified viruses were denatured, loaded and migrated on an SDS page gel. NNV LP2 antigen was detected with a rabbit pAb directed against NNV.

The expression of NNV LP2 antigen by rVHSV_GO_-NNV was assessed in EPC cells. At 24 h post-infection, the expression of the LP2 antigen was evaluated by indirect immunofluorescence on fixed cells (**Figure 6A**). All recombinant viruses expressed the LP2 antigen in infected EPC cells, as shown by the co-labelling with anti-VHSV G mAb and anti-NNV pAb. The incorporation efficiency of the LP2 antigens at the surface of rVHSV_GO_-NNV virions was verified by Western-blot assay on sucrose-purified viruses. **Figure 6B** shows that all rVHSV_GO_-NNV expressed the LP2 antigen at the expected size.

### Safety and protective efficacy of rVHSV_GO_-NNV in rainbow trout

**Figure 7:**
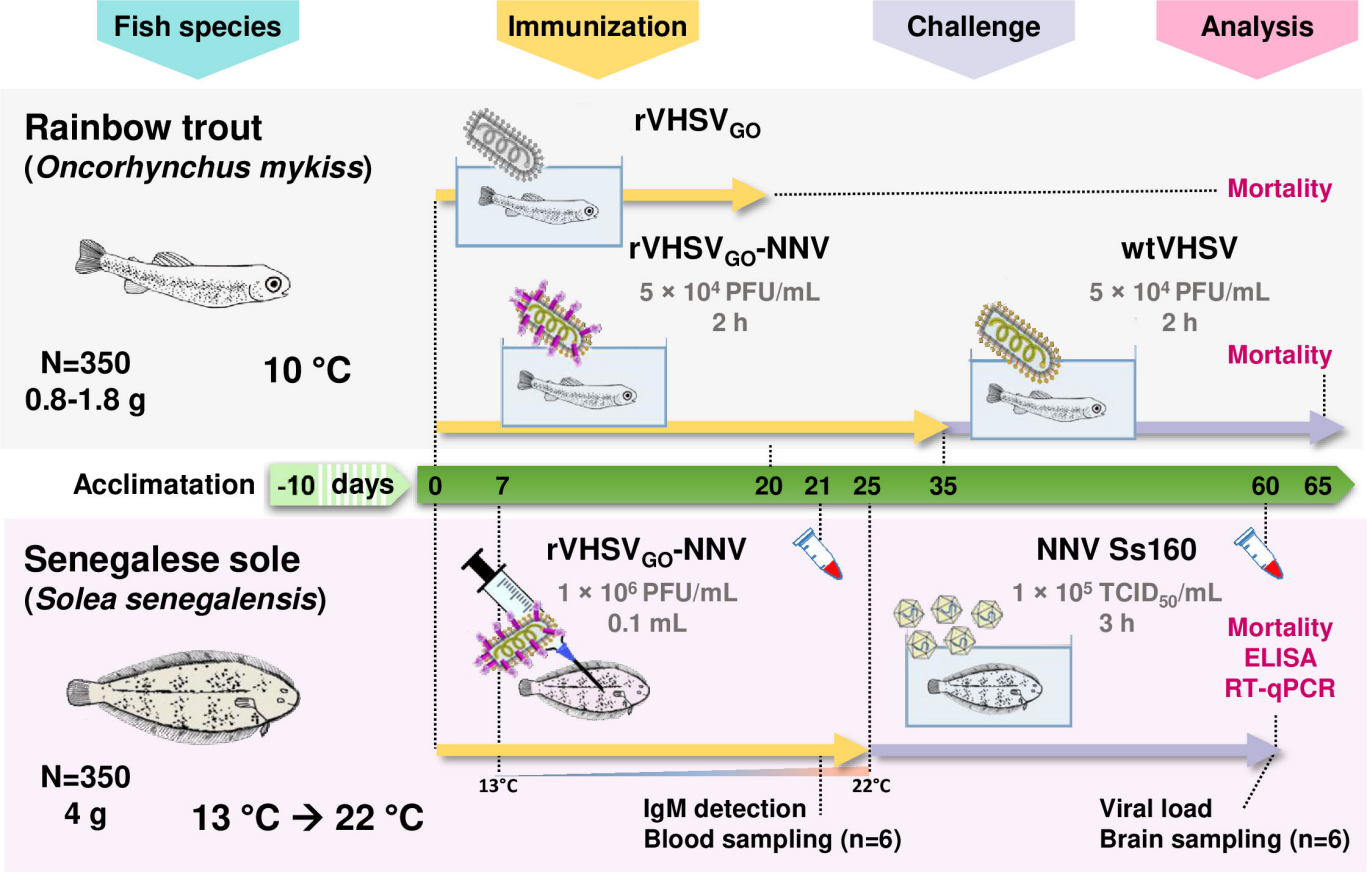
Experimental setup. Three different fish experiments were performed. The first on trout was designed to test the attenuation provided by the gene order rearrangement of the VHSV genome. The second on trout was conducted to test the safety and the protective efficacy of rVHSV_GO_-NNV against a lethal VHSV challenge. All trout infections were performed by bath immersion with a viral load of 5 × 10^4^ PFU/mL in 3 L of water. The third was conducted on sole to test the safety, the immunogenicity and the protective efficacy of rVHSV_GO_-NNV against a lethal NNV challenge. The immunizations and the lethal NNV challenge were performed by injecting 1 × 10^5^ PFU of rVHSV_GO_-NNV per fish and bath immersion with 1 × 10^5^ TCID_50_/mL of NNV, respectively.

**Figure 8:**
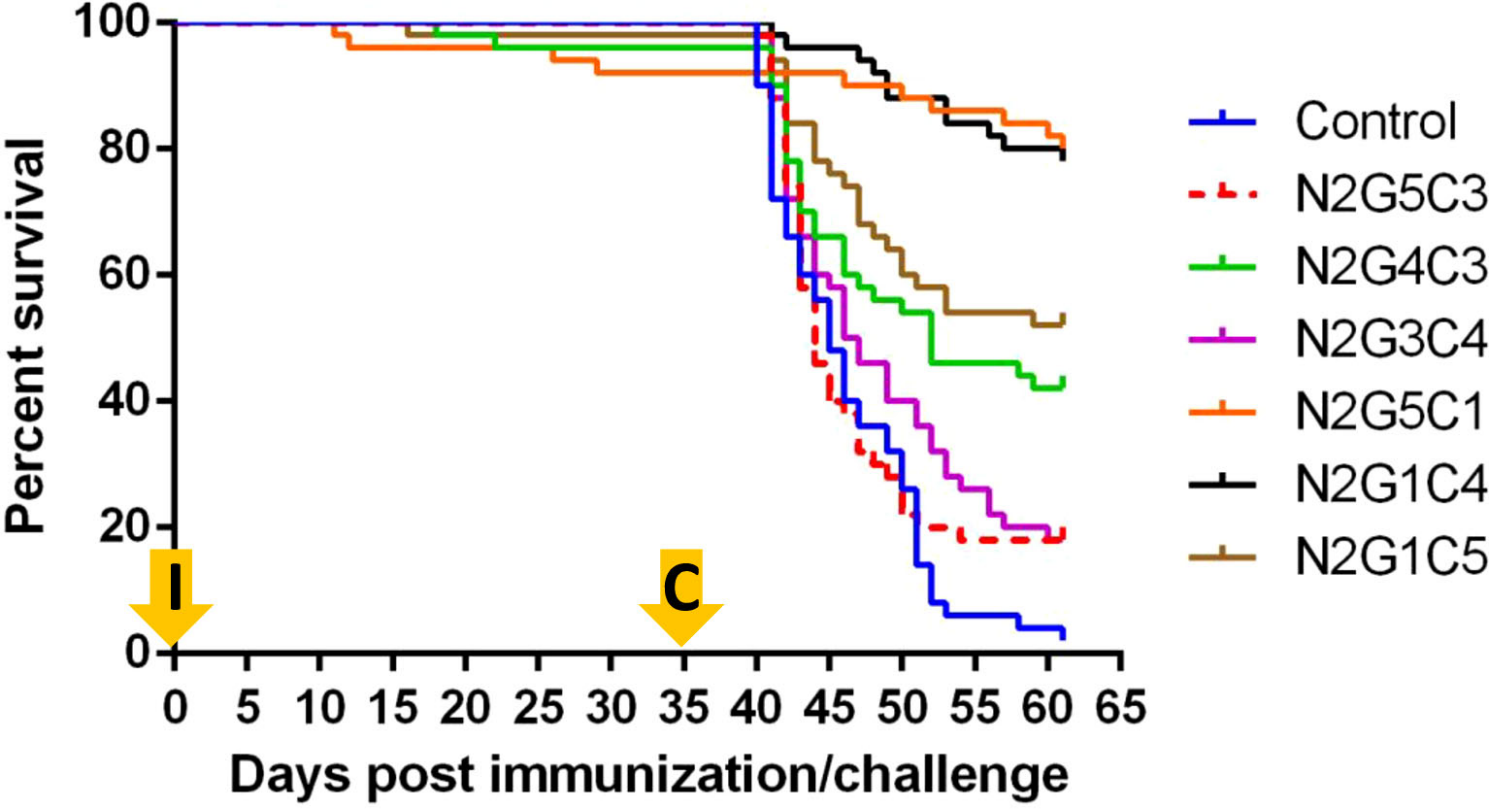
Fish survival curves following infection of trout by bath immersion with rVHSV_GO_-NNV. Fifty virus-free juvenile rainbow trout (mean weight of 0.8 g) were infected with 6 rVHSV_GO_-NNV as described in materials and methods. Fish mortality rates were recorded every day for 35 days. Then, rVHSV_GO_-NNV-immunized fish were challenged with wtVHSV. Fish mortality rates were recorded every day for 30 additional days.

The safety and protective efficacy of the rVHSV_GO_-NNV was assessed by infecting highly sensitive juvenile rainbow trout (mean weight of 0.8 g) and recording mortality rates daily for up to 35 days (**Figure 7**). As shown in **Figure 8**, rVHSV_GO_-NNV were almost completely attenuated. The mortality started at day 11, day 16 and day 18 post infection for N2G5C1, N2G1C5 and N2G4C3, respectively. The residual virulence for these three viruses at day 35 was ranging around 2 to 8% of cumulative mortality. N2G5C3 and N2G1C4 were completely attenuated in juvenile trout. No mortality was recorded for both viruses during the 35-day period, similarly to what is observed with the mock-infected fish control condition.

At 35 days post-immunization, the potential of rVHSV_GO_-NNV as live vaccine was tested by challenging the surviving trout by bath immersion with a lethal dose of wild-type VHSV (**Figure 7**). As shown in **Figure 8**, the mortality in the mock-vaccinated group reached 98% whereas it reached 82% for both N2G5C3 and N2G3C4 groups, 54% for N2G4C3 group, 48% for N2G1C5 group, 22% for N2G1C4 group and 12% for N2G5C1 group. The highest calculated Relative Percent of Survival (RPS) were 78% and 80% for N2G1C4 and N2G5C1, respectively (**Table 1**). In contrast to N2G5C1 inducing 8% of mortality during the immunization step, no mortality was recorded during the immunization for N2G1C4 immunized group. Thus, the overall protection of 78% induced upon vaccination with N2G1C4 by bath immersion makes this virus a promising vaccine candidate.

**Table 1:** Summary of percent cumulative mortality observed in trout infected by rVHSVGO-LinkerP2NNV and challenged by VHSV.

Virus^a^	% cumulative mortality		RPS^d^
	Immunization^b^	Challenge^c^	
**N2G5C3**	0	82	16
**N2G4C3**	4	54	45
**N2G3C4**	0	82	16
**N2G5C1**	8	12	80
**N2G1C4**	0	22	78
**N2G1C5**	0	48	51
**Control** ^e^	0	98	_

a Groups of 50 trout (mean weight of 0.81 g) were immunized by bath immersion with the indicated viruses (5 × 10^4^ PFU/mL).

b Cumulative percent of mortality at day 35 postimmunization.

c VHSV challenge by bath immersion (5 × 10^4^ PFU/mL) was performed at day 35 postimmunization and ended at day 61.

d Relative percent survival (RPS) = 1 - (percent mortality in group/percent mortality in control) × 100 (42).

e Group of fish immunized with virus-free culture medium and challenged with VHSV at day 35 postimmunization.

### Safety, immunogenicity and protective efficacy of rVHSV_GO_-NNV in Senegalese sole

**Figure 9:**
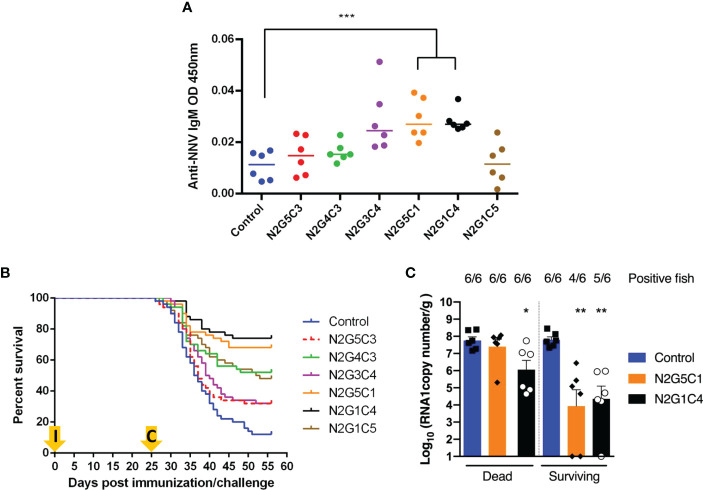
Fish survival curves following immunization by injection of sole with rVHSV_GO_-NNV and challenge with NNV by bath immersion. Fifty virus-free juvenile sole (mean weight of 4 g) were injected with 6 rVHSV_GO_-NNV as described in materials and methods. **(A)** Twenty-one days post infection, six fish per group were sacrificed and blood samples were taken. The levels of anti-NNV antibodies in sera from immunized sole was evaluated by ELISA. **(B)** Fish mortality rates were recorded every day for 25 days. Then, rVHSV_GO_-NNV-immunized fish were challenged with NNV by bath immersion. Fish mortality rates were recorded every day for 31 additional days. **(C)** NNV replication in brain tissues of immunized fish. Six dead fish and six surviving fish randomly harvested at day 30 post-challenge in the indicated groups were analyzed by RT-qPCR. Virus loads are expressed as RNA1 copy per gram of brain. Number of positive fish are indicated above each histogram. For statistical analysis, a comparison between groups was performed with a one-way ANOVA and Tukey’s multiple comparison tests using GraphPad Prism (GraphPad, San Diego, CA). Groups that are significantly different are denoted *(*p* < 0.05), **(*p* < 0.01), ***(p < 0.001).

The safety and immunogenicity of the rVHSV_GO_-NNV was assessed by infecting juvenile sole. Fifty sole (mean weight of 4 g) per group were acclimated at 13°C, the optimal temperature for VHSV replication, and then injected by intra-peritoneal route with 1 × 10^5^ PFU of rVHSV_GO_-NNV per fish (**Figure 7**). 7 days later, the temperature of water in tanks was progressively increased to 22°C, the optimal temperature for NNV replication. After 21 days, six fish per group were sacrificed and blood samples were taken. The levels of anti-NNV antibodies in sera from immunized sole was evaluated by ELISA. As shown in **Figure 9A**, specific and significant antibody responses were detected for two immunized groups: N2G5C1 and N2G1C4. In parallel, the mortality rates were recorded daily for up to 25 days. As shown in **Figure 9B**, rVHSV_GO_-NNV were completely attenuated and safe in sole. No mortality was recorded for all viruses during the 25-day period as for the mock-infected fish.

At 25 days post-immunization when the water temperature in the tanks reached 22°C, the protective efficacy of rVHSV_GO_-NNV was tested by challenging the sole with the wild-type NNV by bath immersion (1 × 10^5^ TCID_50_/mL) (**Figure 7**). As shown in **Figure 9B**, the mortality in the mock-vaccinated group reached 88% whereas it reached 68% for both N2G5C3 and N2G3C4 groups, 52% for N2G1C5 group, 48% for N2G4C3 group, 32% for N2G5C1 group and 26% for N2G1C4 group. The highest calculated RPS were 64% and 70% for N2G5C1 and N2G1C4, respectively (**Table 2**). Both recombinant viruses are associated with considerably reduced NNV load in the brain tissues of surviving fish by almost 10,000-fold compared to non-immunized fish as measured by RT-qPCR (**Figure 9C**) and some of the fish in both groups have no detectable amount of NNV. A significant reduction of NNV load in brain (100-fold compared to control fish) was also observed in dead fish immunized by N2G1C4. The efficient protection induced by these two rVHSV_GO_-NNV was in accordance with the significant antibody titers measured in both immunized groups (**Figure 9A**). Thus, the overall protections of 78% in trout and 70% in sole induced upon vaccination with N2G1C4 make this recombinant and attenuated virus a promising vaccine candidate.

**Table 2:** Summary of percent cumulative mortality observed in sole injected with rVHSVGO-LinkerP2NNV and challenged by NNV.

Virus^a^	% cumulative mortality		RPS^d^
	Immunization^b^	Challenge^c^	
**N2G5C3**	0	68	23
**N2G4C3**	0	48	45
**N2G3C4**	0	68	23
**N2G5C1**	0	32	64
**N2G1C4**	0	26	70
**N2G1C5**	0	52	41
**Control** ^e^	0	88	_

a Group of 50 sole (mean weight of 4 g) were immunized by injection with the indicated viruses (1 × 10^5^ PFU/fish).

b Cumulative percent of mortality at day 25 postimmunization.

c NNV challenge was performed by bath immersion (1 × 10^5^ TCID_50_/mL) at day 25 postimmunization and ended at day 56.

d Relative percent survival (RPS) = 1 - (percent mortality in group/percent mortality in control) × 100 (42).

e Group of 50 fish immunized with virus-free culture medium and challenged with NNV at day 25 postimmunization.

**Figure 10:**
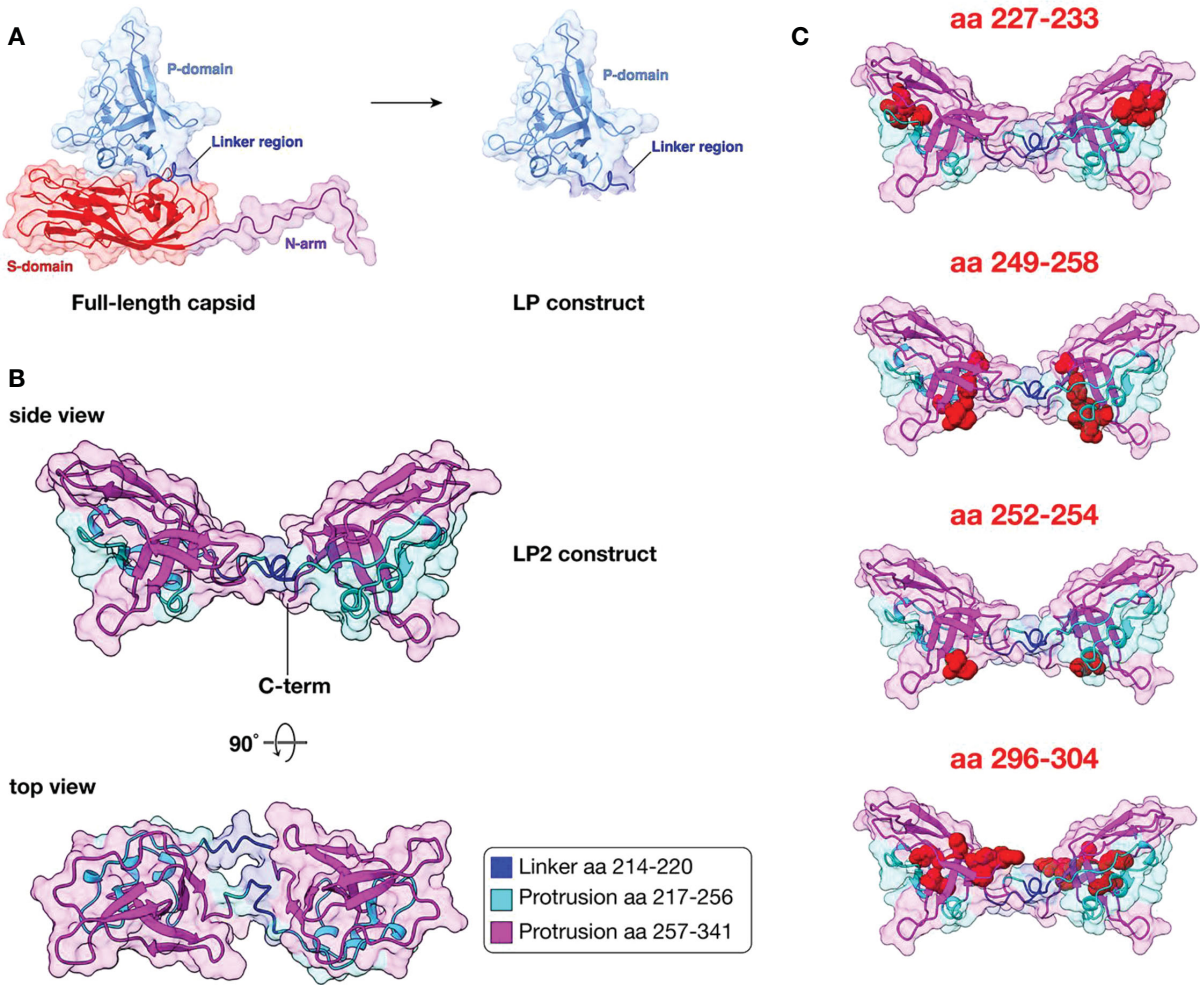
Structural model of the LP2 antigen using AlphaFold 2. **(A)** Structure of nervous necrosis virus (NNV) full-length capsid and Linker-Protrusion (LP) construct. Ribbon diagram with surfaces displayed of NNV capsid subunit and LP construct based on the PDB structure 4WIZ. Molecular visualizations were performed using Chimera X. **(B)** AlphaFold 2 structural model of LP2 construct. The capsid protein sequence (accession no. NC_024493.1) of the betanodavirus isolate SpSsIAusc16003 (related to serotype A SJNNV) was used to generate an AlphaFold 2 structural model using ColabFold. Molecular visualization was performed using UCSF ChimeraX. The structural prediction with highest confidence score (pLDDT of 88.2/100) is shown. The C-terminus of the LP2 construct is shown in the side view to indicate where the TM domain of VHSV is connected (not modeled). The sub-regions of the Linker (L) and Protrusion (P domains) are color-coded according to the color legend on the figure. **(C)** Mapping of known neutralizing epitopes onto LP2 structural model. Confirmed or predicted protrusion domain neutralizing epitopes corresponding to amino acids 227-233, 249-258, 252-254, and 296-304 are highlighted as red spheres. See text for the references describing each epitope.

To gain structural insights into the LP2 construct used in our study, we generated a structural model using AlphaFold 2 based on the protein sequence of the SpSsIAusc16003 isolate, which is a RGNNV/SJNNV reassortant with a capsid protein related to SJNNV (serotype A) (**Figure 10**). The LP2 construct consists of a tandem repeat of the linker (L) and protrusion (P) domains located at the external tip of the viral capsid protein (**Figures 10A, B**). AlphaFold 2 is a powerful deep learning algorithm providing a breakthrough in structural prediction for proteins (43). AlphaFold 2 readily predicts protein structures with atomic accuracy without the need for structural templates, as long as enough orthologs are available for generating multiple sequence alignments enabling covariance evaluation. AlphaFold 2 was able to generate an accurate model of LP2 with an overall confidence score (pLDDT) of 88.2 out of 100, close to the score of 90 corresponding to models with highly confident predictions of both backbone and residue side chain orientations. Viewed from the side, the LP2 model presents a bi-lobed “butterfly” structure of the LP dimer with each lobe consisting of a monomer of the pyramidal protrusion domain connected *via* the linker domain (**Figure 10B**). In the side view, the C-terminus of LP2 is shown to indicate where the VHSV TM domain connects (not modeled). Previous serological analyses of chimeric capsid proteins have shown that the region determining antigenic diversity spans residues 257-341 for the capsid of SJNNV (serotype A) (7). These residues are color-coded onto the modeled structure in magenta (**Figure 10B**). Both side and top views show that the repeated 257-341 region of the protrusion domains are clearly surface-exposed, suggesting that they are readily accessible to neutralizing antibodies (**Figure 10B**). We further mapped onto the LP2 structural model confirmed or predicted protrusion domain neutralizing epitopes (**Figure 10C**). The amino acids (aa) corresponding to the following epitopes were highlighted as red spheres in the LP2 model: aa 227-233 (44); aa 249-258 (45); aa 252-254 (40); and aa 296-304 (46). The mapping suggests that the LP2 construct correctly presents these epitopes at the surface of each LP monomer and would thus allow for antibody binding at these sites.

## Discussion

In the current study, we aimed to generate live-attenuated VHSV vectors expressing NNV major protective antigen in order to characterize their safety, immunogenicity and protective efficacy against these two major diseases for trout and sole aquaculture. Therefore, the VHSV infectious cDNA (27) was modified by rearranging the gene order as a stable attenuation strategy (19, 35, 47) and the addition of an expression cassette driving the insertion of the antigen of interest at the plasma membrane of the infected cells and its incorporation in the newly formed virion (18, 38). Both modifications of the viral genome intrinsically lead to virus attenuation by changing the gradient of viral gene expression, thus several positions of N and G genes together with the expression cassette were tested to find the best combination. Among all rVHSV_GO_-NNV tested here, N2G1C4 presents the best balance between attenuation and protective efficacy. N2G1C4 is safe for both fish species and protects trout and sole against a lethal challenge with VHSV or NNV, respectively. This protection is in accordance with the induction of specific NNV antibodies in sole and should be further tested for its duration. Thus, N2G1C4 represents a promising candidate for the development of a bivalent live attenuated vaccine in order to protect two commercially valuable fish species against infections by these two major pathogens.

One of the challenges in developing live vaccines is to attenuate the virus without substantially reducing its immunogenicity. The genome architecture of *Rhabdoviridae* is highly conserved and viral mRNAs are expressed in a gradient, such that viral proteins at the 3’ proximal end of the viral genome are produced at higher levels than those at the distal end (29, 31). Thus, the rearrangement of gene order has been proposed as an approach to make terrestrial and aquatic rhabdoviruses more suitable as platforms for vaccine development (19, 34) or as candidates for oncolytic virus therapy (48). Homologous recombination appears to be very rare in mononegaviruses (49), making the rearrangement of gene order a safe method to attenuate mononegaviral-derived vectors minimizing the risk of reversion to a virulent phenotype. As previously demonstrated for IHNV, another *Novirhabdovirus*, recombinant viruses with rearranged genomes were highly stable following up to 10 successive passages in fish cells (19).

As previously shown (19), rIHNV N2G3 and N2G4 exhibited slower replication kinetics, reduced viral production (10- to 50-fold reduction compared to the wild-type virus, respectively) and were strong inducer of interferon and interferon stimulated genes (ISGs) in trout cells. More interestingly, they were almost completely attenuated with a residual virulence in juvenile trout (mean weight of 0.7 g) around 15% of cumulative mortality for both viruses versus 90% for the wild-type virus at 35 days post-infection. Trout immunized with rIHNV N2G3 were highly protected against a subsequent infection by a virulent IHNV strain, with RPS from 68% to 86% depending on the mean weight of the fish at the beginning of the experiment (0.7 g and 1 g, respectively). Therefore, we focused first on these two specific gene orders in order to evaluate their respective effect on VHSV virulence in trout. As observed with IHNV, the decrease in N protein expression by moving the N gene in second position in the genome significantly reduced VHSV virulence in trout. Both rVHSV N2G3 and N2G4 were attenuated but a higher residual virulence was observed compared to their IHNV counterparts with a cumulative mortality rate in juvenile trout (mean weight of 1.8 g) of 45% and 57%, respectively, versus 92% for the wild-type virus at 21 days post-infection. These first results represented a solid starting point in order to introduce the expression cassette which was expected have an additional attenuation effect on VHSV. Indeed, the insertion of the expression cassette in position 4 in the N2G3 backbone led to a 10-fold reduction in the final titer of the N2G3C4 (2.5 × 10^8^ PFU/mL versus 2 × 10^7^ PFU/mL), which resulted in total loss of virulence in trout. Unfortunately, the N2G3C4 recombinant virus was found to be over-attenuated as it did not protect immunized fish against a lethal VHSV challenge.

In total six different gene rearrangements were tested in order to find the best balance between attenuation and immunogenicity, since attenuation can result in reduced immunogenicity. With regard to resistance to infection, the protective role of VHSV-neutralizing antibodies has been clearly demonstrated in trout (50). The VHSV glycoprotein G is the neutralization antigen and the major protective antigen. Experimental vaccines designed to induce specific antibodies against VHSV glycoprotein G provide resistance to infection (51–53). One strategy was to move the G gene in the first position and thus increase the expression of this major protective antigen while maintaining the N gene in second position to keep the attenuation effect by reducing the expression of the N protein, leading to N2G1C4 that displays the best balance between attenuation and immunogenicity. This is in accordance with previous studies showing that the expression of the VSV glycoprotein G or the Human respiratory syncytial virus (RSV) fusion F and glycoprotein G could be improved by moving it to a promoter-proximal position (33, 47), while the N gene position was found to be one of the most critical factors regulating rhabdovirus pathogenicity (19, 35). For rIHNV N2G3 and N2G4, lower levels of N transcription were clearly correlated with stronger induction of type I IFN (19). It is possible that a decrease in replication efficiency gives the cell an advantage to mount a strong IFN response, but, this might also be a consequence of the N protein inhibition of the IFN induction pathway, as recently suggested for novirhabdoviruses (54, 55). Therefore, we suggest that higher induction of IFN together with higher expression of the protective antigen contribute to rVHSV N1G1C4 attenuation, immunogenicity and protective immunity in trout and sole.

The NNV antigens that were designed and analyzed in this study are based on the capsid (coat) protein, which is the sole structural protein found in betanodavirus particles. During virion formation multiple capsid proteins self-assemble into a T=3 icosahedral structure, with a total of 180 capsid proteins arranged in 60 trimers (39). As its name implies, the pyramid-like protrusion (P) domain is exposed at the surface of viral particles and harbors both host cell receptor binding and virus neutralization epitope sites. Three serotypes of NNV have been characterized, with SJNNV grouped as serotype A betanodavirus, the cold-water betanodaviruses TPNNV and BFNNV grouping in serotype B, and RGNNV belonging to serotype C (7). Based on antigenic and infectivity assays of the serotype C RGNNV under various physicochemical conditions, Gye and Nishizawa have suggested that the sites responsible for antigenicity and infectivity are distinct (56). In our study, different capsid-derived antigen constructs of the SpSsIAusc16003 isolate, a RGNNV/SJNNV reassortant with a capsid protein related to SJNNV (serotype A) were initially tested, including the linker-protrusion domain (LP) construct (8). The LP2 construct consisting of a tandem repeat of the linker-protrusion domain was then selected and used in subsequent immunization/challenge and ELISA assays. Structural modeling using AlphaFold 2, a highly accurate computational method, revealed that the LP2 construct forms a bi-lobed “butterfly” structure with each lobe consisting of one LP monomer. Each LP monomer exposes a region spanning residues 257-341 which was previously shown to be a determinant of antigenic diversity for the capsid of SJNNV (serotype A) (7). In addition, previously characterized (aa 227-233, aa 249-258, aa 252-254) or predicted (aa 296-304) neutralizing epitopes map to surface-exposed areas of each LP monomer (40, 44–46). Of note, with the exception of the epitope spanning residues 252-254, the so-called “PAN” epitope which corresponds to the three amino acids found in SJNNV (serotype A) capsid, the other previously described epitopes were based on RGNNV (serotype C). Alignment between SJNNV and RGNNV capsid sequences revealed differences in amino acid composition for the 4 epitopes (44). In particular, in their recently published work based on RGNNV (serotype C), Zhang and colleagues mapped the linear epitope _227_SLYNDSL_233_ as the binding site of a monoclonal antibody (Mab 2B7) they produced in mouse and which was shown to be neutralizing. For comparison, the sequence found at residues 227-233 for the SJNNV (serotype A) capsid is _227_PLHNDSI_233_. Interestingly, the authors showed that of the three substitutions found when comparing the 227-233 segment of RGNNV and SJNNV capsids, only the Y229H substitution interfered with MAb 2B7 antibody binding (44). In their discussion, the authors state that pre-immunization of juvenile groupers with the RGNNV 227-233 peptide failed to elicit protective immunity against RGNNV challenge. This result suggests that the native structural context in which the 227-233 segment is presented, such as in our LP2 construct, may be an important contributing factor to obtain immune protection. It is also noteworthy to highlight that another major benefit of our approach using the LP2 construct, where LP is found in duplicate is the possibility to generate an antigen with two protrusion domains derived from two serotypes, for example with serotype A (SJNNV) and serotype C (RGNNV) which are antigenically distinct (7), thus paving the way to the development of a “divalent”-NNV candidate vaccine.

Moreover, Senegalese sole are also susceptible to marine VHSV isolates but are not affected by freshwater isolates, such as the VHSV 23-75 strain used as vaccine vector in the present study (57). Therefore, it should be of great interest to evaluate the level of protection conferred by rVHSV N1G1C4 against infection by marine VHSV strains in immunized sole. Another option would be to pseudotype the rVHSV 23-75 strain with the glycoprotein of a marine strain, novirhabdoviruses being extremely flexible in their capacity to accommodate heterologous glycoproteins (58). This could be the starting point for the development of a bivalent live attenuated vaccine candidate for the protection of senegalese sole against two major diseases. In conclusion, these results validate the gene rearrangement approach as a potent and stable attenuation strategy for fish Novirhabdoviruses and open new perspectives to design a live attenuated vaccine platform for fish vaccinology.

## Methods

### Cells and virus

*Epithelioma Papulosum Cyprini* (EPC) cells were maintained at 24°C in GMEM/HEPES 25 mM medium supplemented with 2 mM L-glutamine (PAA) and with 10% fetal bovine serum (FBS) (Eurobio) (59). rVHSV were propagated in monolayer cultures of EPC cells at 15°C as previously described (27). Virus titers were determined by plaque assays on EPC cells under an agarose overlay (0.35% agarose in Glasgow’s modified Eagle’s medium with 25 mM HEPES supplemented with 2% fetal bovine serum and 2 mM L-glutamine). At 3 to 4 days postinfection, cell monolayers were fixed with 10% formalin and stained with crystal violet. Recombinant vaccinia virus expressing the T7 RNA polymerase, vTF7-3, was kindly provided by B. Moss (National Institutes of Health, Bethesda, Md.) (60).

NNV strain SpSsIAusc16003 (herein Ss160) was grown in E-11 cells, a clone of SSN-1 (61), derived from striped snakehead (*Channa striatus*) at 25°C in L-15 Leibovitz (Lonza) medium supplemented with 2% FBS.

### Virus purification

For virus purification, wild-type and recombinant VHSVs were mass produced in EPC cells, clarified by low-speed centrifugation (4,000 rpm for 15 min), concentrated 10-fold by ultracentrifugation at 24,000 rpm in a SW28 Beckman rotor for 90 minutes and finally purified by ultracentrifugation at 34,000 rpm in a SW41 Beckman rotor for 4 hours through a 25% (w/v) sucrose cushion in TEN buffer (10 mM Tris-HCl [pH = 7.5], 150 mM NaCl, 1 mM EDTA [pH = 8]). The viral pellet was then resuspended in TEN buffer and viral protein yields of each preparation were quantified by using the Micro BCA assay protein quantification Kit (Pierce) in accordance with the manufacturer’s instructions.

Similarly, NNV was grown in a confluent monolayer of E-11 cells maintained in a 150 cm^2^ flask, when the cytopathic effect was extensive, the cell medium was collected and centrifuged at 3,000 × *g* for 10 min at 4°C. The supernatant was centrifuged at 25,000 rpm for 1 h in an SW32Ti rotor (Beckman Coulter). The virus was then pelleted in an ultracentrifuge at 35,000 rpm at 4°C in a SW55Ti rotor (Beckman Coulter) through a 30% (w/v) sucrose cushion in TEN buffer. Pelleted virus was resuspended in TEN buffer for SDS–PAGE.

### Plasmid constructs and recombinant virus recovery

The recombinant cassette integrated into VHSV cDNA between the N and P genes was constructed as previously described (18, 38). The full-length or domains of the NNV capsid gene (GenBank # NC_024493) was amplified by polymerase chain reaction (PCR) from the infectious cDNA encoding RNA2 pBS160 R2 (62) and specifics primers (**Table 3**). Amplified capsid PCR products were cloned into pJET 1.2 plasmid and sequenced to check the integrity of the nucleotide sequence prior to the insertion into pVHSV cassette using *NheI* and *PmlI* enzyme restriction sites (**Figure 1A**).

**Table 3:** Primers used in the study.

Primer	Sequence (5’ to 3’)^a^	Restriction site
5NNV CP	CCCGCTAGCATGGTACGCAAAGGTGATAAGAAATTGG	*NheI*
3NNV CP	GGGCACGTGTTAGTTTTCCGAGTCAACACGGGTG	*PmlI*
5NNV CAP	CCCGCTAGCGTACGCAAAGGTGATAAGAAATTGGC	*NheI*
3NNV CAP	GGGCACGTGGTTTTCCGAGTCAACACGGGTGAAGAGC	*PmlI*
5NNV LP	CCCGCTAGCACACCTGAGGACACCACCGCTCCAATTACTACC	*NheI*
5NNV LP2bis	CCCCACGTGACACCTGAGGACACCACCGCTCCAATTACTACC	*PmlI*
5NHPA	CAAAAGAACTCAGT **GTT** A **AC** ATGGAAGGAGGAATCGTGC	*HpaI*
3NHPA	GACTACCCCGAGGACTCTGACTAA **GTTAAC** CTCCCGTCTCATAACC	*HpaI*
5PPML	GCAAGACAAACACTGAGATCAC **GTG** ATGGCTGATATTGAGATGAGC	*PmlI*
3PPML	GGACAAGCTAGAGTAG **CACGTG** CACAACGCATCACACAG	*PmlI*
5MSNA	GGCAACCAACAACT **T** A **C** G **TA** ATGGCTCTGTTCAAAAGAAAGCG	*SnaBI*
3MSNA	CCTCTGTCCGACCTTGGTAG **TA** CG **T** AAGGACCGACTCAGGC	*SnaBI*
5GBST	GTACACAACAAGCTAGA **GTATAC** ATGGAATGGAACACTTTTTTCTTG	*BstZ17I*
3GBST	CTAGAAGTCAGACGGTCTGAG **T** A **T** ACCTGTCCGAATGACC	*BstZ17I*
5NVPME	GGCACCTTTATGAT **GTTT** AAACATGGCGACCCAACCCGCGC	*PmeI*
3NVPME	GGCTCTGGGCTCACCTCCTGAG **TTTAAA** CGCCGTCTCTCAG	*PmeI*
5VHSN_Spe	ACTAGTATGGAAGGAGGAATTCGTGCAGCG	*SpeI*
3VHSN_Spe	ACTAGTTTAGTCAGAGTCCTCGGGGTAGTCG	*SpeI*
5VHSN_Pml	AGTCACGTGATGGAAGGAGGAATTCGTGCAGCG	*PmlI*
3VHSN_Pml	GAGCACGTGTTAGTCAGAGTCCTCGGGGTAGTCG	*PmlI*
5VHSP_Hpa	GATGTTAACATGGCTGATATTGAGATGAGCGAGTCCTTGG	*HpaI*
3VHSP_Hpa	GTGGTTAACCTACTCTAGCTTGTCCAGCTCCGCC	*HpaI*
5VHSG_Snab	AGATACGTAATGGAATGGAACACTTTTTTCTTGGTGATC	*SnaBI*
3VHSG_Snab	CAGTACGTATCAGACCGTCTGACTTCTAGAGAACTGCTGC	*SnaBI*
5VHSM_BstZ	ACTGTATACATGGCTCTGTTCAAAAGAAAGCGCACC	*BstZ17I*
3VHSM_BstZ	CCTGTATACCTACCAAGGTCGGACAGAGGAGGTTCCAG	*BstZ17I*
5VHSM_Snab	TACGTAATGGCTCTGTTCAAAAGAAAGCGCACC	*SnaBI*
3VHSM_Snab	TACGTACTACCAAGGTCGGACAGAGGAGGTTCCAG	*SnaBI*
5VHSG_BstZ	GTATACATGGAATGGAACACTTTTTTCTTGGTGATC	*BstZ17I*
3VHSG_BstZ	GTATACTCAGACCGTCTGACTTCTAGAGAACTGCTGC	*BstZ17I*
5VHSG_Pml	CACGTGATGGAATGGAACACTTTTTTCTTGGTGATC	*PmlI*
3VHSG_Pml	CACGTGTCAGACCGTCTGACTTCTAGAGAACTGCTGC	*PmlI*
5SP_LP2	ACATACGTAATGGACACCACGATCACCACTCCG	*SnaBI*
3LP2_TM	ATGTACGTATCAGACCGTCTGACTTCTAGAGAACTGCTG	*SnaBI*
5VHSgfpPsi	CGATTATAACAAGACAAACAACTAGTATGGTGAGCAAGGG	*PsiI*
3VHSgfp Psi/Spe	ATTCTTATAATCGTGCCGTTTTTTTCTATCTATGACTAGTTTACTTGTACAGCTCGTCCATGCCG	*PsiI/SpeI*

a. Restriction enzyme sites are underlined; mutated nucleotides are boldfaced.

The different cDNA constructs with rearranged gene order were obtained by gene swap using restriction enzymes (RE), that generate blunt ends, inserted at the beginning and the end of the N, P, M, G, and NV open reading frames (ORF): N (*HpaI*), P (*PmlI*), M (*SnaBI*), G (*BstZ17I*) and NV (*PmeI*) genes (**Figure 4A**). For the insertion of these restriction sites, fragments of the pVHSV (27) were amplified and cloned into pJET 1.2 cloning vectors (Thermo Fischer Scientific) for further site-directed mutagenesis (QuikChange Site-directed mutagenesis Kit, Stratagene) using the primers in **Table 3**: Fragment *SacII/PsiI* (N gene, primers 5NHPA/3NHPA); fragment *PsiI/NsiI* (P gene, primers 5PPML/3PPML); fragment *NsiI/MfeI* (M gene, primers 5MSNA/3MSNA); and fragment *MfeI/NdeI* (G gene, primers 5GBST/3GBST; NV gene, primers 5NVPME/3NVPME). After mutagenesis, all fragments were incorporated back into pVHSV, leading to the pVHSV-RES.

cDNA copies of the N, P, M, and G genes flanked by the proper RE sites were obtained by PCR using the Phusion High-Fidelity DNA polymerase (see primers VHSN, VHSP, VHSM and VHSG with proper RE in **Table 3**) and cloned into pJET 1.2 cloning vector. All cloned genes were sequenced to check the integrity of the nucleotide sequence. Each gene was successively exchanged in the pVHSV-RES, leading to pVHSV N2G4 and pVHSV N2G3 (numbers referring to the positions of the N and G genes in the final cDNA genome) (**Figure 4A**).

In order to construct rearranged VHSV genomes expressing an additional gene (**Figure 5**), the EGFP cassette, previously described (27), was amplified by PCR from the pVHSV-EGFP and modified to contain two *SpeI* RE sites upstream and downstream the EGFP ORF using the primers 5VHSgfp Psi/3VHSgfp Psi/Spe (**Table 3**). This fragment was cloned into a pJET 1.2 cloning vector and then the EGFP ORF was exchanged with the N gene using the *SpeI* RE sites. Finally, the N cassette was inserted into pVHSV-RES using the *PsiI* RE site. Each gene was successively exchanged in the pVHSV-RES containing the expression cassette. Eight pVHSV constructs, termed NxGyCz according to the respective positions of the genes encoding the N and the G as well as the expression cassette C along the genome: pVHSV-N2G5C3, -N2G4C3, -N2G3C4, -N2G3C5, -N2G4C1, -N2G5C1, -N2G1C4 and -N2G1C5.

The rVHSVs were readily recovered by transfection of pVHSV constructs together with the helper plasmids pT7-N, pT7-P and pT7-L in EPC cells infected with vTF7-3 vaccinia virus, as previously described (for a review see (63)). Viral titers were determined after 2 passages on EPC cells.

### Indirect immunofluorescence analysis on fixed and living cells

EPC cells grown in 24-well plates were infected with the rVHSV expressing NNV epitopes (passage 2, MOI of 0.1). At 24 h or 72 h post-infection, cells were fixed with a mixture of ethanol and acetone (1:1, v/v) at -20°C for 20 min and washed with PBS. Primary mouse monoclonal antibody (mAb) 192A17 (dilution 1:1,000) against VHSV G and rabbit polyclonal antibody (pAb) 484.2.2009 against NNV (dilution 1:5,000; kindly provided by Dr. Anna Toffan (7)) were incubated in PBS-Tween 0.05% for 45 min at room temperature (RT) and washed 3 times with PBS-Tween 0.05%. Cells were then incubated with Alexa Fluor 488-conjugated goat anti-mouse and Alexa Fluor 594-conjugated goat anti-rabbit immunoglobulins diluted to 1:1,000 (Invitrogen) in PBS-Tween 0.05% for 45 min at RT. Cell monolayers were then visualized with a UV-light microscope (Carl Zeiss). For live cells, infected cell monolayers were directly incubated with primary antibodies in GMEM 10% FBS cell culture medium for 45 min at RT. After 3 washes with the same medium, cells were incubated with both 488 and 594 Alexa Fluor-conjugated immunoglobulins (dilution 1:1,000) for 45 min at RT. Three washes were performed and nuclei were stained with Hoechst (dilution 1:1,000; Thermo scientific). Cell monolayers were then visualized with a UV-light microscope (Carl Zeiss).

### Protein electrophoresis and Western blot assays

Aliquots of sucrose-purified recombinant viruses were separated on a sodium dodecyl sulfate 4-12% polyacrylamide gel (SDS-PAGE; Life technologies) and electrotransferred onto a polyvinylidene difluoride membrane (ImmobilonP; Millipore). The membrane was saturated in Tris-Buffer Saline containing 0.05% of Tween 20 (TBST) supplemented with 5% skim-milk for 1 h at RT, then incubated with a rabbit pAb 484.2.2009 in TBST 5% milk (dilution 1:7,000) for 1 h at RT. After three washes with TBST, the membrane was incubated for 1 h at RT with horseradish peroxidase (HRP) conjugated anti-rabbit antibody (1:10,000; Sera Care) in TBST 3% milk. After extensive washing with TBST, peroxidase activity was revealed by incubation with ECL Western Blotting Detection Reagents (ECL; Pierce) according to the manufacturer’s instructions.

### Ethics statement

All animal studies were carried out in strict accordance with the European guidelines and recommendations on animal experimentation and welfare (European Union directive 2010/63). All animal experiment procedures were approved by the local ethics committee on animal experimentation (COMETHEA INRAE no. 45) and were authorized by the Ministère de l’Éducation nationale, de l’Enseignement supérieur et de la Recherche under the numbers: APAFIS#2545-2015121515466368 v1 and APAFIS#29801-2021021110262075 v2. Experimental protocols with sole were approved by the Bioethics and Experimental Animal Welfare Committees of the University of Santiago de Compostela and Xunta de Galicia (Permit Id. 15010/2020/004).

To minimize animal suffering and distress, all manipulations were carried out under light anesthesia. Anesthesia was performed by bath immersion with tricaine 0.005%. A lethal challenge with VHSV typically results in acute disease characterized by exophthalmia, anemia and punctiform hemorrhages, whilst NNV infection lead to anemia, and abnormal swimming due to neurological disorders. Therefore, fish were monitored twice a day for clinical signs and survival. Upon display of typical infection symptoms, animals were humanely euthanized by bath immersion using a lethal dose of tricaine 0.015%.

### Experimental fish infection

As summarized in **Figure 7**, 50 INRA synthetic strain virus-free juvenile rainbow trout (mean weight, 0.8 to 1.8 g) were infected by immersion in tanks filled with 3 L of freshwater with rVHSV viruses (final titer, 5 × 10^4^ PFU/mL) for 2 h at 10°C. Tanks were then filled up to 30 L with freshwater. Controls were mock infected fish kept under the same conditions. Mortalities were recorded daily. Challenges with wild-type VHSV were performed under similar conditions 35 days after immunization. Senegalese sole (~4 g, on average) were acclimatized to 13°C (immunization temperature) for 10 days in our facilities prior to immunization (see **Figure 7**). After the acclimation period, fish were gently sedated with MS-222 and injected intraperitoneally with 0.1 mL of mutant rVHSV viruses (1 × 10^6^ PFU/mL) and kept in 5 L opaque tanks containing seawater (n = 50/tank). After 7 days the water temperature was increased 1°C/day to reach 22°C (challenge temperature) on day 25. Blood samples (n = 6 per group) were taken 21 days post-immunization and the surviving fish were challenged by immersion in a bath containing the lethal Ss160.03 NNV strain at a concentration of 10^5^ TCID_50_/mL for 3 h with strong aeration. Mortalities and clinical signs were recorded daily. Control fish were mock immunized/infected with L-15 medium under the same conditions. Brains from six dead (at different time post-challenge) or surviving fish were aseptically collected in each group. The organs were individually homogenized and diluted 1 : 10 (w/v) in Earle’s balanced salt solution (Hyclone) supplemented with penicillin (1000 UI ml−1), streptomycin (1000 µg ml−1), gentamicin (500 µg ml−1) and fungizone (20 µg ml−1). The homogenates were clarified by centrifugation at 2000 g for 20 min at 4°C. An aliquot of 0.1 ml of each sample was used for RNA extraction, and subjected to RT-qPCR as described previously (64). Viral load data were calculated as RNA1 copies per gram of brain tissue.

### Indirect ELISA for anti-betanodavirus antibody analyses

The level of anti-betanodavirus antibodies in sera from immunized sole have been evaluated following the indirect ELISA procedure previously reported (65). Briefly, sera from sole immunized with the different recombinant viruses (20 µg of total proteins) were diluted in coating buffer [100 mM Bicarbonate/Carbonate, pH 9.6] and immobilized in 96 High Binding flat-bottomed plates (Sarsted, Newton, NC, USA) overnight at 4°C. The samples were blocked with 5% skimmed milk in PBST for 1 h. Afterwards, incubation with a rabbit anti-NNV (484.2.2009, 1:10,000) was performed for 1 h at room temperature. Following washing steps, the samples were incubated with the anti-rabbit IgG-HRP (Sigma Aldrich; 1:25,000) for 1 h at RT. The reaction was revealed with 100 µL per well of 3,3’,5,5’-tetramethylbenzidine single solution (ThermoFisher, Waltham, MA, USA) for 20 min and stopped by adding 50 µL of 2 M sulfuric acid. Optical density (OD) was measured at 450 nm. Resulting OD values were normalized by subtracting the OD values of the negative control (omitting fish sera) wells. All assays were performed in duplicate and previously assayed positive serum was used as a positive control.

### Structural prediction of LP2 antigen construct using AlphaFold 2

The protein sequence (accession no. NC_024493.1) encoding the capsid of the betanodavirus isolate SpSsIAusc16003 was used to generate structural predictions of the LP2 antigen construct using AlphaFold 2 (43). The open-source software, ColabFold (https://github.com/sokrypton/ColabFold) was used to implement AlphaFold 2 (66). The predicted structure with the highest confidence score (pLDDT) was subsequently used for molecular visualization using UCSF ChimeraX (67).

## Data availability statement

The original contributions presented in the study are included in the article/supplementary material. Further inquiries can be directed to the corresponding authors.

## Ethics statement

The animal study was reviewed and approved by COMETHEA INRAE no. 45 and the Ministère de l’Éducation nationale, de l’Enseignement supérieur et de la Recherche under the numbers: APAFIS#2545-2015121515466368 v1 and APAFIS#29801-2021021110262075 v2 and by Bioethics and Experimental Animal Welfare Committees of the University of Santiago de Compostela and Xunta de Galicia (Permit Id. 15010/2020/004).

## Author contributions

SS, EM, MB, JM and SB conceived and designed the experiments. SS, EM, AC, AL and JB performed the experiments. SS, JM and SB analyzed the data. SS, JM and SB wrote the paper. All authors contributed to the article and approved the submitted version.
